# Circular RNA hsa_circ_0007142 Is Upregulated and Targets miR-103a-2-5p in Colorectal Cancer

**DOI:** 10.1155/2019/9836819

**Published:** 2019-06-26

**Authors:** Chang-li Zhu, Xiaofeng Sha, Yuan Wang, Jin Li, Men-yan Zhang, Zhong-ying Guo, Su-an Sun, Jing-dong He

**Affiliations:** ^1^Department of Medical Oncology, Huai'an First People's Hospital, Nanjing Medical University, Huai'an City, Jiangsu Province 223300, China; ^2^Department of Oncology, People's Hospital of Hongze District, Huai'an City, Jiangsu Province 223300, China; ^3^Department of Pathology, Huai'an First People's Hospital, Nanjing Medical University, Huai'an City, Jiangsu Province 223300, China

## Abstract

Circular RNAs (circRNAs) are a large class of endogenous noncoding RNAs that regulate gene expression and mainly function as microRNA sponges. This study aimed to explore the aberrant expression of circRNAs in colorectal cancer (CRC). Using a circRNA microarray, we identified 892 differentially expressed circRNAs between six pairs of CRC and adjacent paracancerous tissues. Among them, hsa_circ_0007142 was significantly upregulated. Further analysis in 50 CRC clinical samples revealed that hsa_circ_0007142 upregulation was associated with poor differentiation and lymphatic metastasis of CRC. Bioinformatic analysis and luciferase reporter assay showed that hsa_circ_0007142 targeted miR-103a-2-5p in CRC cells. Moreover, the silencing of hsa_circ_0007142 by siRNAs decreased the proliferation, migration, and invasion of HT-29 and HCT-116 cells. Taken together, these findings suggest that hsa_circ_0007142 is upregulated in CRC and targets miR-103a-2-5p to promote CRC.

## 1. Introduction

Colorectal cancer (CRC) has the third highest incidence among cancers and is the fourth leading cause of cancer-related mortality worldwide [1]. Although several treatment options have achieved remarkable advances, the survival of CRC patients remains unsatisfactory, mainly due to the lack of effective biomarkers for stratification in personalized treatment. With the advent of precision medicine and next generation sequencing, it is urgent to identify valuable biomarkers for the diagnosis, prognosis, and therapy of CRC.

Circular RNAs (circRNAs) are noncoding RNAs that play an important role in regulating gene expression and function [2]. Compared with regular linear RNAs, circRNAs are produced with covalently linked ends, leading to increased stability [3]. Accumulating data have shown that circRNAs are aberrantly expressed in many tumors [4–8]. CircRNAs mainly serve as microRNA (miRNA) sponges to regulate miRNAs, such as the interaction of CDR1as with miR-7 in cervical carcinoma [9], the sponge function of circRNA-100338 for miR-141-3p in hepatocellular carcinoma (HCC) [10], and circRNA_100269 as the sponge for miR-630 in gastric cancer (GC) [11]. In particular, dysregulated circRNAs play important role in CRC via the regulation of multiple miRNAs [12, 13]. Furthermore, circRNAs could serve as transcriptional enhancers or competitors with linear splicing to regulate host gene expression in multiple diseases [14–17]. Many circRNAs have been reported to be potential biomarkers of CRC [18]. However, the function of these dysregulated circRNAs in CRC remains to be further investigated. In our previous study, we performed a circRNA microarray analysis with a panel of six CRC tissues and six normal tissues [19]. In this study, we focused on circRNA hsa_circ_0007142 because it showed the second highest upregulation in CRC in previous study [19]. We first confirmed the upregulation of hsa_circ_0007142 in CRC tissues by RT-qPCR analysis and analyzed the association of hsa_circ_0007142 with clinicopathological aspects of CRC. Next we investigated the functional role of hsa_circ_0007142 in the proliferation, migration, and invasion of CRC cells.

## 2. Materials and Methods

### 2.1. Tissue Specimens and Clinical Data

The present study was approved by the Ethics Committee of Huai'an First People's Hospital, Nanjing Medical University (Huai'an, China), and written informed consent was obtained from each patient. The tissue samples of CRC and paired adjacent paracancerous tissues were obtained from CRC patients at Huai'an First People's Hospital. All patients did not receive prior radiotherapy and chemotherapy, and CRC was confirmed by experienced pathologists. After surgery, the tissues were quickly snap-frozen and stored at -80°C until further analysis.

### 2.2. Cell Culture

Human colorectal cancer HCT-116, HT-29, and LoVo cells and normal human enteral epithelial (HCO) cells were obtained from the Shanghai Institutes for Biological Sciences (Shanghai, China) and cultured in Roswell Park Memorial Institute (RPMI) 1640 medium (Hyclone, Logan, UT, USA) supplemented with 10% FBS, 100 units/mL of penicillin, and 100 mg/mL of streptomycin at 37°C in an incubator with 5% CO_2_.

### 2.3. Reverse Transcription Quantitative PCR (RT-qPCR)

Total RNA was isolated from CRC tissues and cells using TRIzol reagent as described previously [20]. For circRNA analysis, cDNAs were synthesized with 1 *μ*g of total RNA using random primers. For miRNAs, specific primers were used for cDNA synthesis. The expression levels of circRNAs or mRNA were determined using RT-qPCR, with GAPDH as an internal control. For miRNA expression, U6 was used as an internal control for normalization. All experiments were independently conducted in triplicate, and the 2^−ΔΔCT^ method was used to analyze the data. The classification of CRC patient grouping was derived from the value of 2^−ΔΔCT^ and the cut-off of the hsa_circ_0007142-high group was set as 1. The sequences of the primers are listed in Table 1.

### 2.4. Cell Transfection

The control and si-hsa_circ_0007142 siRNAs (mixtures of three siRNAs targeting different sites of hsa_circ_0007142) were designed and synthesized by RiboBio (Guangzhou, China; Table 1). HT-29 and HCT116 cells were seeded in six-well plates at a density of 5×10^5^/well and cultured to a confluency of 50-60% and then were transfected with control or gene specific siRNAs using Lipofectamine 2000 (Invitrogen, Carlsbad, CA, USA).

### 2.5. Cell Proliferation Assay

The Cell Counting Kit-8 (CCK-8, Dojindo, Japan) assay was performed to evaluate CRC cell proliferation. Briefly, 48 h after transfection, cells were seeded in 96-well plates at a density of 5×10^3^/well, and cell viability was measured on 1, 2, 3, and 4 days. CCK-8 reagent was added into each well and maintained at 37°C for 2 h. The OD value at 450 nm was measured using a microplate reader (Bio-Rad, Hercules, CA, USA).

### 2.6. Colony Formation Assay

Transfected cells were seeded into 6-well plates and cultured for 15 days. Then the cells were fixed with methanol and stained with 0.5% crystal violet (Beyotime Biotechnology) for 30 min. Colonies with more than 10 cells were counted under a light microscope.

### 2.7. Cell Migration and Invasion Assay

A 24-well Transwell insert with 8 *μ*m pore (Corning, NY, USA) was used for the cell migration and invasion assays. 48 h after transfection, 1×10^5^ cells were plated in the upper chamber and 500 *μ*l of medium was added to the lower chamber for the migration assay. For the invasion assay, the upper layer of membrane inserts was added with 40 *μ*g of Matrigel per well and incubated at 37°C for 1 h. After 48 h, cells that migrated to the lower compartment through the coated membrane were fixed with methanol, stained with 1% crystal violet, and quantified under a microscope. All assays were performed in triplicate.

### 2.8. Luciferase Assay

The plasmids carrying the fragment of either the wild-type or the mutant hsa_circ_0007142 sequence of the predicted miR-103a-2-5p binding sites were constructed by Geneseed (Guangzhou, China). HT-29 cells were cotransfected with the plasmids and miR-103a-2-5p mimic using Lipofectamine 2000 (Invitrogen, Carlsbad, CA, USA). After 48 h, cells were collected and luciferase activity was determined using the Dual Luciferase Reporter Assay System (Promega, Madison, WI, USA).

### 2.9. Statistical Analysis

All data were presented as mean ± standard deviation (SD) and analyzed using SPSS 20.0 software (IBM, USA). Differences between groups were analyzed using Student's* t*-test, Wilcoxon test, or* X*^*2*^*-*test. A two-tailed* P*-value <0.05 was considered statistically significant.

## 3. Results

### 3.1. hsa_circ_0007142 Is Upregulated and Correlates with the Clinicopathological Features of CRC Patients

**Figure 1:**
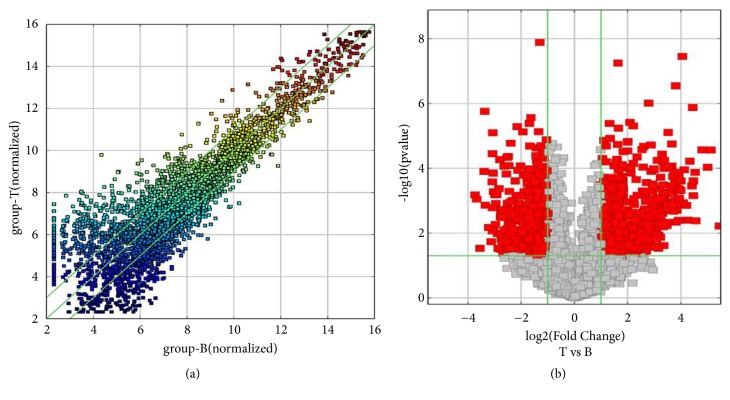
Comparison of circRNA expression profiles between CRC and paired pericancerous tissues. (a) Scatter plots for assessing the difference in the expression of circRNAs among samples (T for CRC and B for adjacent tissues). The values of each group were plotted on the X and Y axes (log⁡2 scaled). The middle green line represented no difference between two groups, while the flank green lines indicated 2-fold changes. The circRNAs beyond the lines referred to > 2-fold changes between two groups. (b) Volcano plots for differential circRNA expression between two groups. The vertical lines indicated 2 folds (log⁡2 scaled), and the horizontal line represented* P* of 0.05 (log⁡10 scaled). The red dots represented differentially expressed circRNAs with statistical significance.

To assess the expression of circRNAs in CRC, the differential expression of circRNAs between six CRC tissues and six paracancerous tissues was examined by circRNA microarray. A total of 892 differentially expressed circRNAs were found in CRC, including 412 upregulated and 480 downregulated (fold >2,* P*<0.05; Figures 1(a) and 1(b)). The top 10 upregulated and downregulated circRNAs are listed in Table 2. The five potential target miRNAs of each of the top 10 upregulated and downregulated circRNAs were predicted by Arraystar software according to TargetScan and miRanda database (Table 3). Based on the expression level, the specificity of primers, the efficiency of siRNA-interference, and the function of potentially targeted miRNAs, we chose hsa_circ_0007142 derived from linear RNA DOCK1 for further investigation.

**Figure 2:**
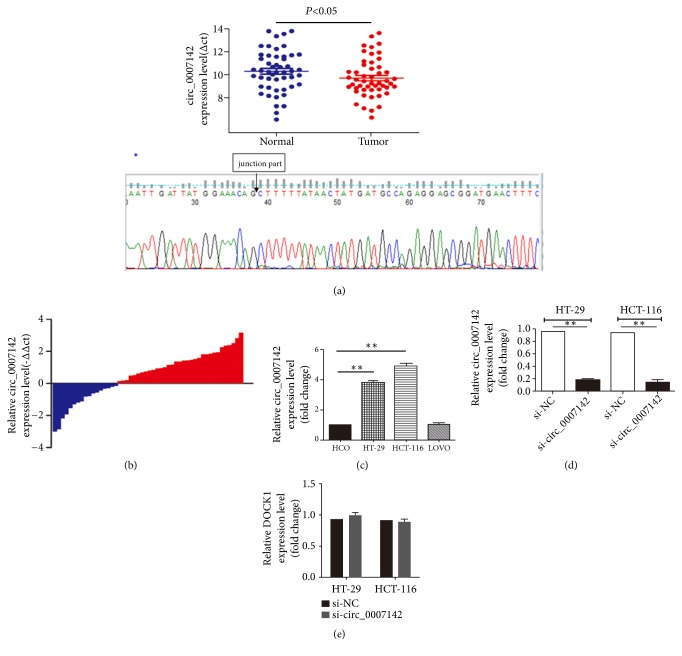
hsa_circ_0007142 expression is elevated in CRC tissues and cells. (a) hsa_circ_0007142 expression in CRC tissues (*n*=50) and paired pericancerous tissues (*n*=50) was determined by PCR and normalized to GAPDH. Sequencing results confirmed PCR products, especially the junction part. (b) The tissues were divided into two groups. Positive –ΔΔcT indicated high hsa_circ_0007142 expression, while negative -ΔΔCT indicated low hsa_circ_0007142 expression. (c) Relative hsa_circ_0007142 expression in HT-29, HCT116, LoVo, and HCO cells. (d) Relative hsa_circ_0007142 expression level in HT-29 and HCT-116 cells transfected with circ_0007142 (si-circ_0007142) or control siRNA (si-NC). (e) Relative DOCK1 mRNA expression in HT-29 and HCT116 cells transfected with si-circ_0007142 or si-NC. Error bars represented mean ± standard deviation (SD). *∗P<*0.05 and *∗∗P<*0.01. ΔCT is the difference between the CT value of hsa_circ_0007142 and CT value of GAPDH. ΔΔCT is the difference between the ΔCT of CRC tissues and the ΔCT of adjacent normal tissues.

In order to validate the upregulation of hsa_circ_0007142 in CRC, we examined hsa_circ_0007142 expression level by RT-qPCR in 50 clinical CRC specimens and pericancerous tissues. Compared to adjacent noncancerous tissues, CRC tissues had a significantly higher hsa_circ_0007142 expression (*P*<0.05, Figures 2(a) and 2(b)). Sequencing results of PCR products confirmed the expression of hsa_circ_0007142 (Figure 2(a)). In addition, we analyzed the association of hsa_circ_0007142 expression with CRC clinical features and found that high expression of hsa_circ_0007142 was associated with the differentiation (*P*=0.008) and lymphatic metastasis of CRC (*P*=0.037) (Table 4). These results suggest the important role of hsa_circ_0007142 in the progression of CRC.

### 3.2. hsa_circ_0007142 Promotes the Proliferation of CRC Cells

**Figure 3:**
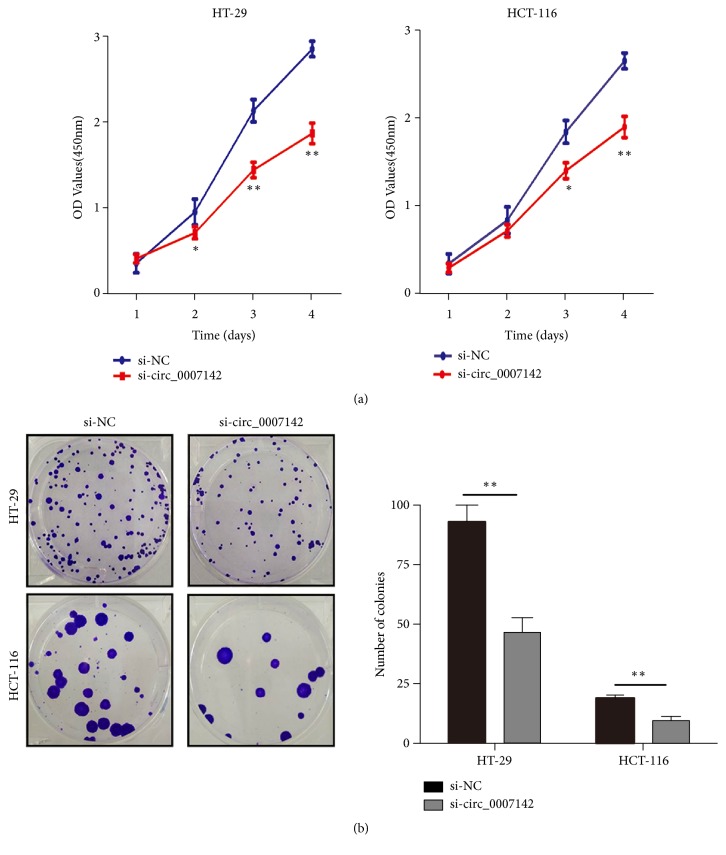
hsa_circ_0007142 knockdown inhibited CRC cell proliferation. (a) CCK8 assay of the viability of HT-29 and HCT116 cells transfected with circ_0007142 (si-circ_0007142) or control siRNA (si-NC). (b) Colony-forming assay of HT-29 and HCT-116 cells transfected with si-circ_0007142 or si-NC. Data were presented as the mean ± SD of three independent experiments. *∗P<*0.05, *∗∗P<*0.01.

To explore the role of hsa_circ_0007142 in CRC, first we compared its expression level in CRC cell lines HCT-116, HT-29, and LoVo with normal enteral epithelial cell line HCO. The expression of hsa_circ_0007142 was significantly higher in HCT-116 and HT-29 cells than in HCO cells, but there was no significant difference between HCO and LoVo cells (Figure 2(c)). Next, loss-of-function assays were performed by transfecting specifically designed siRNAs or negative control siRNA into HT-29 and HCT-116 cells. We found that hsa_circ_0007142 siRNA significantly decreased hsa_circ_0007142 in both HCT-116 and HT-29 cell lines (Figure 2(d)). In order to exclude the possibility of siRNAs abating DOCK1, which produces hsa_circ_0007142 during the transcription, the expression level of DOCK1 mRNA was examined. As expected, no difference in DOCK1 expression was observed between HCT-116 and HT-29 cells transfected with hsa_circ_0007142 and negative control siRNAs (Figure 2(e)). CCK-8 assay showed that hsa_circ_0007142 knockdown by siRNA significantly inhibited the proliferation of HCT-116 and HT-29 cells in a time dependent manner (Figure 3(a)). In addition, colony formation assay showed that the colony forming ability of HCT-116 and HT-29 cells transfected with hsa_circ_0007142 siRNA was significantly reduced compared to cells treated with control siRNA (Figure 3(b)). Collectively, these results suggest that hsa_circ_0007142 promotes the proliferation of CRC cells.

### 3.3. hsa_circ_0007142 Enhances the Migration and Invasion of CRC Cells

**Figure 4:**
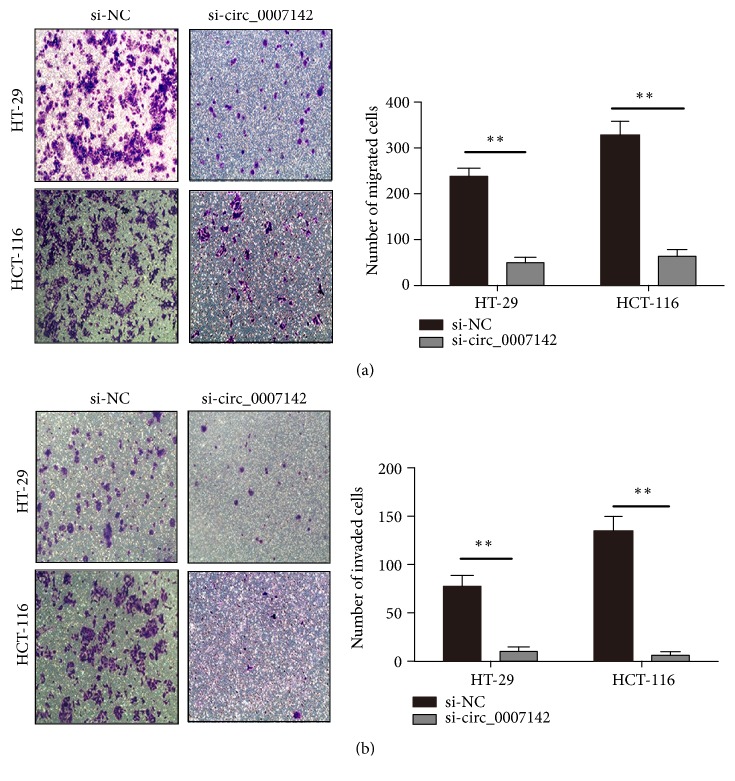
hsa_circ_0007142 knockdown inhibited CRC cell migration and invasion. Transwell assays on the migration (a) and invasion (b) of HT-29 and HCT-116 cells transfected with si-circ_0007142 or si-NC. *∗∗P<*0.01; magnification ×100.

Since hsa_circ_0007142 upregulation was associated with lymphatic metastasis of CRC, next we investigated the role of hsa_circ_0007142 in the migration and invasion of CRC cells. Chamber assay showed that, compared to control siRNA groups, the knockdown of hsa_circ_0007142 by siRNA effectively decreased the invasion (Figure 4(a)) and migration (Figure 4(b)) of both HCT-116 and HT-29 cells.

### 3.4. MiR-103a-2-5p Is the Direct Target of hsa_circ_0007142

**Figure 5:**
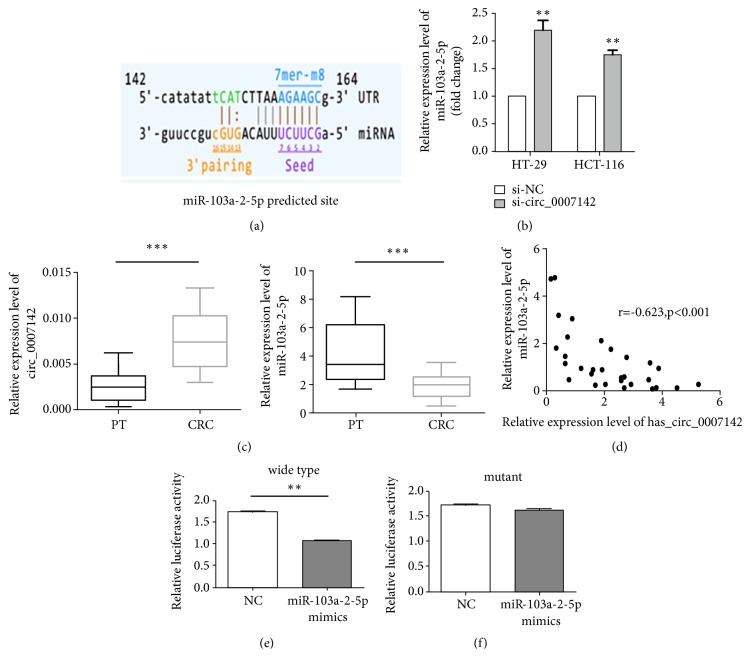
MiR-103a-2-5p is a target of hsa_circ_0007142. (a) The binding site of miR-103a-2-5p for hsa_circ_0007142 was predicted by circRNA microarray. (b) miR-103a-2-5p expression was determined by RT-qPCR in HT-29 and HCT-116 cells transfected with si-circ_0007142 or si-NC. (c) PCR analysis of hsa_circ_0007142 and miR-103a-2-5p expression in CRC tissues (*n*=30) and paired pericancerous tissues (PT,* n*=30). (d) The correlation of the expression of miR-103a-2-5p and hsa_circ_0007142 in CRC tissues (*n*=30) and paired pericancerous tissues (*n*=30). (e) The luciferase activity in HT-29 cells cotransfected with the plasmids of wild-type hsa_circ_0007142 and miR-103a-2-5p mimics. (f) The luciferase activity in HT-29 cells cotransfected with plasmids of mutant hsa_circ_0007142 and miR-103a-2-5p mimics. *∗P<*0.05, *∗∗P<*0.01.

Arraystar software revealed that hsa_circ_0007142 might target miR-103a-2-5p (Figure 5(a)). As expected, significant increase in miR-103a-2-5p expression was found in cells transfected with hsa_circ_0007142 siRNA (Figure 5(b)). Next, the expression of miR-103a-2-5p and hsa_circ_0007142 in 30 pairs of CRC and adjacent noncancerous tissues was detected by RT-qPCR. We found that the expression of hsa_circ_0007142 was significantly upregulated while the expression of miR-103a-2-5p was significantly downregulated in CRC tissues compared to paired pericancerous tissues (PT) (Figure 5(c)). Moreover, the expression of miR-103a-2-5p was negatively correlated with hsa_circ_0007142 expression (*P*<0.01, r = -0.623, Figure 5(d)). Finally, luciferase reporter assay showed that luciferase activity significantly decreased in HT-29 cells cotransfected with the plasmids of wild-type hsa_circ_0007142 and miR-103a-2-5p mimics, compared to cells cotransfected with plasmids of mutant hsa_circ_0007142 and miR-103a-2-5p mimics (Figure 5(e)). In contrast, there was no significant difference in luciferase activity in HT-29 cells cotransfected with miR-103a-2-5p mimics and plasmids of mutant hsa_circ_0007142, where the miR-103a-2-5p binding site was mutated (Figure 5(f)). Taken together, these results demonstrate that hsa_circ_0007142 targets miR-103a-2-5p in CRC cells.

## 4. Discussion

As endogenous noncoding RNAs, circRNAs exhibit a remarkable organization- and disease-specific characteristic, suggesting that circRNAs may serve as specific biomarkers for disease diagnosis and therapy [21]. A negative relationship between the universal reduction of circRNAs and CRC cell proliferation was observed [21]. Furthermore, circRNA ITCH inhibited esophageal squamous cell carcinoma via regulating Wnt/*β*-catenin pathway [22]. Moreover, circRNAs are involved in epithelial-mesenchymal transition [23]. Numerous studies have revealed that circRNAs play a crucial role in the initiation and progression of cancers through multiple mechanisms [24]. The well-studied function of circRNAs is acting as miRNA sponge to insulate miRNAs and terminate the regulation of their target genes [9, 25]. For example, circHIPK3, as a sponge of miR-124, could upregulate miR-124 target AQP3 expression in hepatocellular carcinoma [26]. Furthermore, circDOCK1 has been reported to inhibit the function of miR-196a-5p by binding miR-196a-5p and enhancing the expression of BIRC3 in OSCC [27]. With the rapid advance of deep RNA sequencing, an increasing number of aberrantly expressed circRNAs have been identified. However, the function of circRNAs in the initiation, progression, and maintenance of different cancers is far from being understood.

In present study, we performed circRNA microarray analysis and identified 892 differentially expressed circRNAs in CRC tissues versus paracancerous tissues. We focused on a distinctly upregulated circRNA hsa_circ_0007142, which was confirmed to be upregulated in CRC tissues and cells. The silencing of hsa_circ_0007142 by siRNAs decreased the proliferation, migration, and invasion of CRC cells. Importantly, hsa_circ_0007142 upregulation was correlated to the differentiation and lymphatic metastasis of CRC. These findings suggest that hsa_circ_0007142 might play an oncogenic role in CRC.

Furthermore, bioinformatics predicted that miR-103a-2-5p was a target of hsa_circ_0007142. While several studies suggested oncogenic role of miR-103a in CRC [28, 29], it was reported that miR-103a could inhibit gastric cancer cell proliferation, migration, and invasion [30]. In addition, miR-103a-2-5p had the capability to inhibit cell survival and colony formation by targeting PARP-1 [31]. Nevertheless, the role of miR-103a-2-5p in CRC remains unknown up to date. In this study we employed luciferase assay to confirm that miR-103a-2-5p was a target of hsa_circ_0007142. It should be pointed out that circRNAs usually target several miRNAs. For example, circRNA ITCH acted as the sponge of miR-7, miR-17, and miR-214 to inhibit esophageal squamous cell carcinoma [22]. Further studies are needed to identify other potential targets of hsa_circ_0007142 and better understand the role of hsa_circ_0007142 in CRC.

In summary, the expression of hsa_circ_0007142 is upregulated in CRC tissues and is associated with the differentiation and lymphatic metastasis of CRC. Furthermore, hsa_circ_0007142 promotes the proliferation and invasion of CRC probably by functioning as a sponge for miR-103a-2-5p. These findings may provide new clue for the strategies in the diagnosis and therapy of CRC.

## Figures and Tables

**Table 1:** The sequences for primers and siRNAs.

Primers used for qRT-PCR		
GAPDH F		GGGAGCCAAAAGGGTCAT
GAPDH R		GAGTCCTTCCACGATACCAA
hsa_circ_0007142 F		GAACTCTGCCTCAGGATGAA
hsa_circ_0007142 R		AACGTGTAACCTCGGTACCA
U6	F	CTCGCTTCGGCAGCACA
U6	R	AACGCTTCACGAATTTGCGT
U6	RT	GTCGTATCCAGTGCAGGGTCCGAGGTAT
		TCGCACTGGATACGACCAAATATGGAAC
miR-103a-2-5p	F	GCGCGAGCTTCTTTACAGTGCT
miR-103a-2-5p	R	ATCCAGTGCAGGGTCCGAGG
miR-103a-2-5p	RT	GTCGTATCCAGTGCAGGGTCCGA
		GGTATTCGCACTGGATACGACCA AGGC
siRNAs oligonucleotides		
si-circ_0007142		GGAAACAGCTTTTTATAAC

**Table 2:** The top ten upregulated and downregulated circRNAs in CRC ranked by fold changes in microarray data.

CircRNA ID	CircRNA type	Chrom	Gene symbol	Fold change	Regulation	P value
hsa_circ_0000072	exonic	chr1	OMA1	43.4572991	up	0.005985797
hsa_circ_0007142	exonic	chr10	DOCK1	34.9353568	up	2.71168E-05
hsa_circ_0008812	exonic	chr9	RAD23B	32.0813361	up	0.00009244
hsa_circ_0050514	exonic	chr19	UBA2	27.7446201	up	2.68425E-05
hsa_circ_0085923	exonic	chr8	PLEC	23.5020249	up	0.004162563
hsa_circ_0087855	exonic	chr9	RAD23B	23.161069	up	0.000133271
hsa_circ_0005954	exonic	chr6	AMD1	22.1403464	up	0.00069505
hsa_circ_0005050	exonic	chr2	XPO1	22.0953662	up	0.000244329
hsa_circ_0005281	exonic	chr17	TBCD	21.7534364	up	1.32686E-06
hsa_circ_0000994	exonic	chr2	SLC8A1	21.3375077	up	0.000346993
hsa_circ_0072279	exonic	chr5	NUP1	13.3423298	down	0.000659178
hsa_circ_0001704	intragenic	chr7	-	12.7785132	down	0.000891495
hsa_circ_0035626	exonic	chr15	RPS27L	11.6798313	down	0.029341895
hsa_circ_0001525	intronic	chr5	-	10.49926	down	0.000123931
hsa_circ_0005904	exonic	chr2	DCAF17	10.3297308	down	1.74038E-06
hsa_circ_0008367	exonic	chr9	IARS	10.183079	down	0.00136856
hsa_circ_0047303	exonic	chr18	ZNF521	9.2977975	down	0.006717343
hsa_circ_0087565	exonic	chr9	ZNF169	8.7255455	down	0.018358601
hsa_circ_0000488	intronic	chr13	-	8.4844294	down	0.000260793
hsa_circ_0031569	exonic	chr14	HEATR5A	8.432469	down	0.00854826

CircRNA ID: The circRNA ID can be found in circBase (http://www.circbase.org/). Gene symbol represents the liner RNA where

circRNAs generated from. “-” indicates that circRNA microarray did not provide the gene symbol of circRNA derived from intragenic or intronic types.

Fold Change: calculated from the ratio of the two groups (normal tissues versus CRC tissues). P-value: computed from paired t-test.

**Table 3:** Five target miRNAs of differentially expressed circRNAs in microarray data.

CircRNA ID	Regulation	MRE
hsa_circ_0000072	up	hsa-miR-136-5p, hsa-miR-606, hsa-miR-145-5p, hsa-miR-153-5p, hsa-miR-302d-5p
hsa_circ_0007142	up	hsa-miR-651-3p,hsa-miR-103a-2-5p,hsa-miR-744-3p,hsa-miR-96-3p,hsa-miR-128-3p
hsa_circ_0008812	up	hsa-miR-138-5p,hsa-miR-325,hsa-miR-593-3p,hsa-miR-512-3p,hsa-miR-766-5p
hsa_circ_0050514	up	hsa-miR-433-3p,hsa-miR-215-3p,hsa-miR-34b-5p,hsa-miR-23b-3p,hsa-miR-891a-5p
hsa_circ_0085923	up	hsa-miR-580-5p,hsa-miR-198,hsa-miR-656-5p,hsa-miR-219a-5p,hsa-miR-758-5p
hsa_circ_0087855	up	hsa-miR-138-5p,hsa-miR-325,hsa-miR-593-3p,hsa-miR-512-3p,hsa-miR-766-5p
hsa_circ_0005954	up	hsa-miR-520f-3p,hsa-miR-618,hsa-miR-223-3p,hsa-miR-875-3p,hsa-miR-495-3p
hsa_circ_0005050	up	hsa-miR-452-5p,hsa-miR-146b-5p,hsa-miR-146a-5p,hsa-miR-597-3p,hsa-miR-578
hsa_circ_0005281	up	hsa-miR-576-3p,hsa-miR-646,hsa-miR-219a-2-3p,hsa-miR-181b-2-3p,hsa-miR-181b-3p
hsa_circ_0000994	up	hsa-miR-27b-3p,hsa-miR-27a-3p,hsa-miR-373-5p,hsa-miR-335-3p,hsa-miR-628-5p
hsa_circ_0072279	down	hsa-miR-665,hsa-miR-874-5p,hsa-miR-188-3p,hsa-miR-29b-2-5p,hsa-miR-1271-3p
hsa_circ_0001704	down	hsa-miR-20b-3p,hsa-miR-641,hsa-miR-766-3p,hsa-miR-661,hsa-miR-625-5p
hsa_circ_0035626	down	hsa-miR-619-3p,hsa-miR-653-5p,hsa-miR-660-3p,hsa-miR-548d-5p,hsa-miR-200a-3p
hsa_circ_0001525	down	hsa-let-7i-5p,hsa-let-7g-5p,hsa-miR-619-5p,hsa-let-7f-5p,hsa-miR-98-5p
hsa_circ_0005904	down	hsa-let-7g-5p,hsa-let-7i-5p,hsa-miR-98-5p,hsa-miR-141-3p,hsa-miR-545-3p
hsa_circ_0008367	down	hsa-miR-330-5p,hsa-miR-216a-3p,hsa-miR-342-5p,hsa-miR-891a-3p,hsa-miR-326
hsa_circ_0047303	down	hsa-miR-149-3p,hsa-miR-17-3p,hsa-miR-1301-3p,hsa-miR-509-5p,hsa-miR-516b-5p
hsa_circ_0087565	down	hsa-miR-766-3p,hsa-miR-612,hsa-miR-338-3p,hsa-let-7g-3p,hsa-let-7a-2-3p
hsa_circ_0000488	down	hsa-miR-519a-3p,hsa-miR-519b-3p,hsa-miR-28-5p,hsa-miR-130b-3p,hsa-miR-708-5p
hsa_circ_0031569	down	hsa-miR-128-3p,hsa-miR-30c-2-3p,hsa-miR-216a-3p,hsa-miR-585-5p,hsa-miR-140-3p

MRE: miRNA response element.

**Table 4:** Correlation between hsa_circ_0007142 expression and clinicopathological characteristics of CRC.

Clinical Parameter	Hsa_circ_0007142 (2^−ΔΔCT^)		Chi-squared test
	High group	Low group	P value
	(≥1, n=33)	(<1, n=17)	
Gender			0.623
Male	21	12	
Female	12	5	
Age (years)			0.209
≤50	6	7	
50–70	15	6	
> 70	12	4	
Tumor size			0.577
≤3 cm	8	5	
3–5 cm	17	10	
>5 cm	8	2	
Differentiation			0.008*∗∗*
Poor	2	6	
Moderate	24	11	
Well	7	0	
Lymphatic metastasis			0.037*∗*
Nx&N0	13	12	
N1&N2	20	5	
Tumor stage			0.136
I–II	19	6	
III–IV	14	11	
Distant metastasis			0.11
No	33	15	
Yes	0	2	

*∗P<*0.05, *∗∗P<*0.01. ΔCT is the difference between the CT value of hsa_circ_0007142 and of GAPDH.

ΔΔCT is the difference between the ΔCT of CRC tissues and the ΔCT of adjacent normal tissues.

## Data Availability

All data are available upon request.
